# Reflections on the Multidisciplinary Health Force: much done, but more to do

**DOI:** 10.1093/pubmed/fdac097

**Published:** 2022-11-21

**Authors:** Frank Houghton

**Affiliations:** Technological University of the Shannon, Department of Applied Social Sciences, Limerick, Ireland

Wright *et al*. are to be commended for providing a factual, historical and albeit rather staid, detailed account of the development of the multidisciplinary public health workforce in the UK.^1^ These authors ask if the work is ‘almost there?’ The assumption being that the work is perhaps effectively complete and that this is a reasonable question. This commentary is an unapologetically situated, opinionated and deliberately provocative response. My aim is to challenge the Faculty of Public Health (FPH) and other Public Health leaders to critically evaluate current practices, trends, and its wider responsibilities and opportunities to drive and define Public Health leadership globally. This response will also explore potential impacts of the coronavirus disease of 2019 (COVID-19) pandemic on developments in the multidisciplinary public health (MDPH) workforce into the future.

An obvious starting point in any discussion of this issue must be to explore evidence around the introduction of formal, recognized training and career opportunities for MDPH personnel in the UK. What are the strengths and limitations of these developments? At one level it seems odd to even have to be raising this question at this point. Not that it is not a valid or pertinent question, but rather the opposite; it seems almost impossible to imagine that such a profound change in the training and delivery of Public Health could even have been contemplated without the inclusion of formal evaluation structures in place. Snippets of evidence have emerged during this process,^2–4^ but systematic evidence and evaluation is both sadly, and glaringly, lacking.

Wright *et al.* state that ‘eight years on, it is timely to review the position with regard to the multidisciplinary endeavour’.^1^ This deficit is astounding for a field in which evaluation is so strongly engrained as to be a foundation of practice.

Tables 1 and 2 detail essential Public Health functions as routinely discussed in the UK and the USA.^5^^,^^6^ It is clear that as well as workforce development, evaluation is acknowledged as a routine and important part of public health. Perhaps a crucial deficit is the absence of an explicit focus on evaluation in relation to workforce development in the (UK) Faculty of Public Health list of functions.

**Table 1:** Functions of the national, regional and local public health system^5^

Health protection	Public health intelligence
Outbreak prevention and control Emergency planning Risk management Infection control Outbreak management Monitoring threats Immunization	Health needs Health outcomes Analysis Information sharing Quality
**Health improvement**	**Academic public health**
Needs assessment Programme development Partnership working Community development Advocacy Sustainability Evidence and evaluation	Advocacy Research Application of public health evidence **Evaluation** Teaching
**Health services**	**Workforce development**
Health service commissioning Health and social care service prioritization Equity Quality **Evaluation** Safety Healthcare development Leadership	Leadership Capacity building Specialty training

**Table 2:** Ten essential public health services^6^

(i) Assess and monitor population health status, factors that influence health, and community needs and assets (ii) Investigate, diagnose and address health problems and hazards affecting the population (iii) Communicate effectively to inform and educate people about health, factors that influence it, and how to improve it (iv) Strengthen, support and mobilize communities and partnerships to improve health (v) Create, champion, and implement policies, plans and laws that impact health (vi) Utilize legal and regulatory actions designed to improve and protect the public’s health (vii) Assure an effective system that enables equitable access to the individual services and care needed to be healthy (viii) Build and support a diverse and skilled public health workforce (ix) Improve and innovate public health functions through ongoing evaluation, research and continuous quality improvement (x) Build and maintain a strong organizational infrastructure for public health

It is hard not to see echoes of early comments around developments in UK MDPH with Evans & Knight having previously highlighted ‘There was no plan!’.^7^ Even the most basic cycle of evaluation developed by Deming in the 1950s codified the Plan-Do-Check-Act (PDCA) cycle.^8^ However, even such basic steps appear to have been omitted in formal developments in the training and functioning of the Public Health workforce. It is too easy to dismiss the absence of such evaluations on the basis that developments in MDPH were political.^9^ Practitioners in Public Health do not need to see quotes from Virchow (‘politics is nothing else but medicine on a large scale’) to be aware of the political nature of all of Public Health.^10^ The FPH need to press for a rigorous evaluation of the MDPH project in a formal, structured, objective, ongoing and transparent manner. To ensure its independence, this evaluation should be commissioned and funded by an external body. The Wellcome Trust or the UK’s National Institute for Health and Care Research (NIHR) would be obvious candidates to commission and oversee such research. This research would necessarily also include an evaluation of the UK Public Health Register (UKPHR), which was a vital development in the professional recognition of MDPH professionals.

Wright *et al*. and others have also highlighted the gradual sifting process, which is evident in employment patterns with medically qualified Public Health personnel often migrating into more lucrative roles within Public Health England, whereas other members of the MDPH workforce being employed in less lucrative local government roles.^1^^,^^4^^,^^11^ This process is hugely problematic for the MDPH project. Even more problematic is the lack of overt, vocal and sustained action by the FPH and other Public Health leaders to highlight and respond to such developments. The influence of employers in any such process is important, but the FPH and allied groups need to engage more substantively in tackling this issue.

As a conflict theorist, I find it impossible not to see this sifting process as suiting some of the more traditional, medically trained personnel within Public Health. Accounts by Wright *et al*. and others have clearly outlined resistance to MDPH by medically trained Public Health personnel, as well as internal strife within the medical bloc as Public Health personnel there fought for recognition and consultant status.^1^^,^^4^ The development of an unofficial de facto two-tier Public Health system by the back door is little more than the ‘occupational protectionism’ that has overtly marked Public Health in the past.^12^ Such dichotomous development will, if unchecked, result in undermining MDPH. The positive gains made to date are at risk.

The gradual re-introduction of such a two-tier hierarchical system has important implications for the effective functioning of Public Health. Tables 1 and 2 clearly demonstrate the multitude of tasks facing Public Health. The diverse competencies required to respond to such challenges have been outlined in the UK and elsewhere.^13–15^ Public Health requires crucial skill sets that extend far beyond the narrow confines of medicine and Public Health medical training. As such formal MDPH training programs, combined with equitable, attractive and fulfilling career structures, with appropriate remuneration, are essential to the long-term success of Public Health. The sifting process may, in time, return the MDPH workforce to the ‘ghetto’,^16^ whereas Public Health medicine enjoys its more lucrative and elevated position.

It is crucial to remember that the diversity in the Public Health skill-mix required mirrors the importance of diversity in Public Health workforce itself. The unfortunate reality is that medicine in the UK, and elsewhere remains lacking in diversity across a range of important facets, including social class.^17–20^ This lack of appropriate insider knowledge, experience and understanding has been referred to as the second prevention paradox, i.e. ‘that prevention measures are often developed by individuals outside of the population in question and may offer little actual benefit to that population’.^21^ MDPH with its range of disciplines and entry routes is less elite than medicine and provides a greater degree of much needed diversity into the field of Public Health.

Although there is now widespread support for the development of MDPH in the UK, the training program itself is extremely limited in terms of numbers. The continuing inequalities between medical and non-medical Public Health specialists are significant. However, equally significant inequalities exist between the privileged few who are selected for Public Health specialty training and the much larger group of practitioners who often have to make do with 'do-it-yourself' training. Such practitioners face a very real glass ceiling, which makes it extremely unlikely they will ever progress to Specialist status. Prof. Maggie Rae, immediate past-President of the FPH, alluded to this recently, stating that ‘we have more than 10 applications for each training post from people who fit the eligibility criteria’.^22^ There is significant need to provide further opportunities for professional development opportunities for this crucial workforce. Action and advocacy by the FPH and Public Health leaders on this issue is urgently required.

The developments in MDPH training and career structures are extremely significant on an international scale. The role of the FPH in the development of MDPH has been crucial. Despite some resistance among medically qualified staff in Public Health the UK has developed a pioneering and innovative model that has the potential to positively influence the development of Public Health training and structures globally. The limited formal development of MDPH training and employment structures elsewhere demonstrates how important developments in the UK have been.^23^

As well as working in Public Health in Ireland, I have also worked in academic Public Health in the USA (Eastern Washington University), and applied Public Health in New Zealand (Tairawhiti District Health). I have therefore witnessed at first hand the US model of Public Health, which is innately multidisciplinary by nature, and the New Zealand Public Health system where leadership of Public Health Units is not restricted to medically qualified personnel. Nonetheless, the UK system developed by the FPH alongside the Department of Health remains a global pioneer and leader.

There is now a clear need for the FPH to build on their innovations and achievements. The FPH must now use their influence and power to effect similar developments in other areas and jurisdictions. At this point this even includes Northern Ireland. Northern Ireland remains the one country within the UK, which has also not included MDPH candidates in its Public Health specialty training programme. Consultant Public Health posts in Northern Ireland are still restricted to medically trained Public Health professionals. The FPH are complicit in such inequalities by continuing to support training schemes in this jurisdiction.

Similarly, examining the UK’s closest neighbor, Ireland, the FPH recognizes membership and fellowship of the Faculty of Public Health Medicine in Ireland. However, reciprocal recognition only applies to medically trained personnel. There is a relatively high degree of movement between the two systems in medical circles. The FPH therefore has a certain degree of influence that it has to date chosen not to exert. MDPH in Ireland therefore remains in the ‘ghetto’, with Public Health in Ireland remaining outdated, hierarchical and medically dominated.^16^ These are increasingly considered outdated occupational protectionist practices and the FPH and Public Health leaders should consider distancing themselves from such and develop a professional public health workforce fit for the 21st Century.

A vocal campaign to encourage the introduction of MDPH in Ireland through journals^24–28^ and newsletters^29–44^ was met by silence from the Faculty of Public Health Medicine of Ireland (FPHMI). For reasons of occupational protectionism the FPHMI continue to ignore McPherson *et al*.’s important statement: ‘Public health needs to be led by genuine, knowledgeable, lifetime and committed enthusiasts, from whatever background’.^45^ The FPH should urgently engage in further dialogue with other Public Health systems and consider removing recognition of training in other jurisdictions that do not actively support, develop, and facilitate MDPH.

My final area of concern relates to the future trajectory of Public Health. Examinations of Public Health functions through the COVID-19 pandemic have identified both strengths and limitations. The need for increased cooperation, joint working and multidisciplinary approaches has been clear in many appraisals. My real concern is that this detail will be lost in popular consciousness and political will and direction over time. Despite the breadth of skill-mix required to respond to the pandemic my fear is that COVID-19 will be remembered as the viral threat that was defeated by medical and biomedical interventions alone. In the public’s mind Public Health may be reduced to biomedical approaches. This mis-perception will over time impact funding priorities and the pipelines into the profession. This may suit more traditional sections of the Public Health workforce and lead to further sifting of the workforce between those with medical training and those without discussed above. The FPH must redouble its emphasis on the need for MDPH and underline the crucial importance of broad skill-mix required to adequately protect and promote Public Health.

Much has been achieved in the 50 years since the creation of the Faculty of Public Health Medicine (FPHM). One of its most significant steps has been to embrace MDPH and become instead the Faculty of Public Health (FPH). The immediate past-President of the FPH, Prof. Maggie Rae is another example of progress within the FPH. Prof Rae is herself from a MDPH background. However, much work remains to be done. There is a need to evaluate not only the training, but the effectiveness of the MDPH project in the workplace. Such examinations should include staff from all backgrounds, including medicine. The FPH must also directly explore and vocally and constructively respond to the apparent sifting process in employment patterns in Public Health that threaten to deliver a dichotomous hierarchical system that could in time destroy the MDPH project. The FPH must also develop its leadership on an international stage and cease to recognize training in other jurisdictions that privilege medicine to the exclusion of other elements of the MDPH team. Finally the FPH should work to ensure that in public consciousness the COVID-19 pandemic does not medicalize Public Health. Public Health is wider than pandemic preparedness, and even within that field, a full spectrum of disciplines and their unique skills are required.

## Notes

**Frank Houghton**, Director of the Social Sciences ConneXions research institute at TUS
